# Analysis of Kinase Gene Expression Patterns across 5681 Human Tissue Samples Reveals Functional Genomic Taxonomy of the Kinome

**DOI:** 10.1371/journal.pone.0015068

**Published:** 2010-12-03

**Authors:** Sami Kilpinen, Kalle Ojala, Olli Kallioniemi

**Affiliations:** Institute for Molecular Medicine Finland (FIMM), University of Helsinki, Helsinki, Finland; The German Cancer Research Center, Germany

## Abstract

Kinases play key roles in cell signaling and represent major targets for drug development, but the regulation of their activation and their associations with health and disease have not been systematically analyzed. Here, we carried out a bioinformatic analysis of the expression levels of 459 human kinase genes in 5681 samples consisting of 44 healthy and 55 malignant human tissues. Defining the tissues where the kinase genes were transcriptionally active led to a functional genomic taxonomy of the kinome and a classification of human tissues and disease types based on the similarity of their kinome gene expression. The co-expression network around each of the kinase genes was defined in order to determine the functional context, i.e. the biological processes that were active in the cells and tissues where the kinase gene was expressed. Strong associations for individual kinases were found for mitosis (69 genes, including *AURKA* and *BUB1*), cell cycle control (73 genes, including *PLK1* and *AURKB*), DNA repair (49 genes, including *CHEK1* and *ATR*), immune response (72 genes, including *MATK*), neuronal (131 genes, including *PRKCE*) and muscular (72 genes, including *MYLK2*) functions. We then analyzed which kinase genes gain or lose transcriptional activity in the development of prostate and lung cancers and elucidated the functional associations of individual cancer associated kinase genes. In summary, we report here a systematic classification of kinases based on the bioinformatic analysis of their expression in human tissues and diseases, as well as grouping of tissues and tumor types according to the similarity of their kinome transcription.

## Introduction

Much of our knowledge on the functions of genes, both in health and disease, is derived from molecular biological experiments with specific model systems, which often provide a biased and context-specific view of gene functions. In *in vivo* in human tissues, gene function often varies from one organ to another as well as across different disease states. The 518 human kinases represent an intensively studied class of proteins which may regulate the activity of up to a third of all human proteins by phosphorylation [1]. Because of their central role in cell signalling, kinases are important targets for drug development, particularly in cancer [2], [3], [4], [5]. The role of kinase genes in cancer has been systematically studied at the genomic DNA level. For example, a recent cancer gene census [6] lists 33 kinases that may undergo genetic alterations in cancer. Numerous reports have also been published on the differential expression of kinase genes and proteins in specific types of cancers, but these results are highly biased towards the most commonly studied kinase genes and towards common cancer forms. Furthermore, published data are often contradictory, precluding an assessment of the overall importance of kinases across different diseases. Kinase genes are known to be strongly regulated at the protein level, but transcriptional level regulation has not been comprehensively studied.

Meta-analyses of large publicly available microarray data sources, such as GeneExpressionOmnibus [7] and ArrayExpress [8] have been shown to facilitate the analysis gene expression across healthy and disease states [9], [10], [11]. However, due to the variability of microarray platforms from one study to another, most investigators have analyzed each of the multiple datasets separately [12], [13], [14], [15], [16], [17], focusing on e.g. cancer-normal comparisons within a tissue type. We reasoned that a direct systematic comparison of kinase gene expression levels across all cells, tissues and disease states would be more informative. We have previously developed and reported a methodology for integrating very large quantities of human expression data into a unified format in order to create a comprehensive reference database of the human transcriptome, GeneSapiens [18]. Here, we made use of this methodology in order to perform the first systematic study of kinase gene expression levels and co-expression networks across thousands of healthy and malignant tissues.

## Results

### Definition of the transcriptional activity of kinase genes

**Figure 1:**
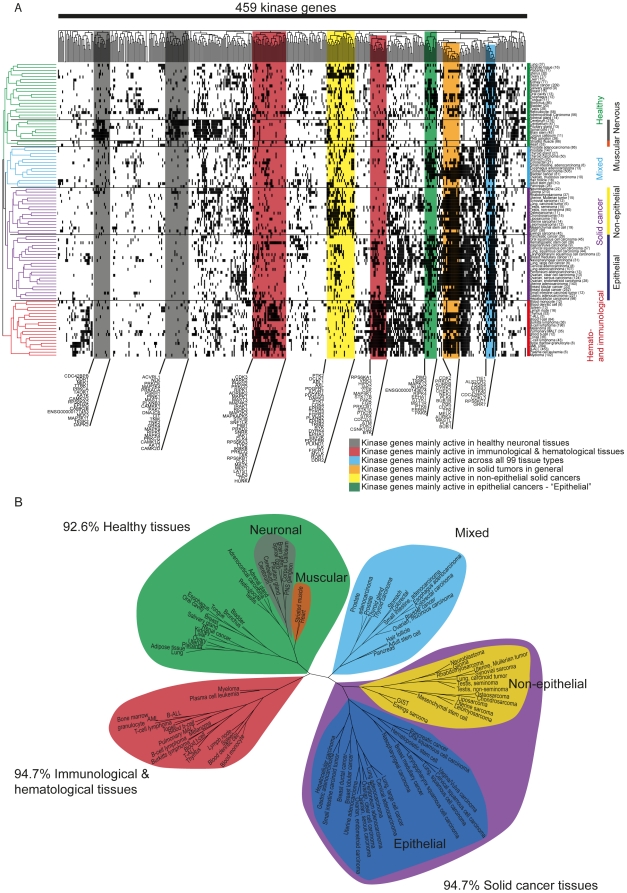
A) Kinase transcriptional activity over 44 healthy and 55 malignant tissues. The number of samples per tissue is given in parentheses. The x-axis contains 459 kinase genes. Black indicates transcriptional activity of the kinase in the tissue. The figure has been clustered in both dimensions (binary distance measure with complete linkage). Several tissue groups can be identified (marked as color bars on the right side of the image). Correspondingly several groups of kinases can be identified having a distinctly different activity profile in tissue groups (colored vertical bars, with kinase gene names of the identified groups shown below). **B) Tree of tissues as defined their by transcriptionally active kinome (same as on the left side of image in panel A).** The four main groups of tissues are mainly solid healthy tissues (92.6%), immunological & hematological (94.7%), solid cancer tissues (94.7%) and a mixed one. Within these groups there are some more specific clusters like neuronal and muscular on the healthy side and non-epithelial and epithelial on the cancer side. Epithelial cancers also show visible tendency to cluster to adeno and squamous groups according to their transcriptionally active kinome.

The analysis focused on 459 genes encoding proteins with protein kinase activity (Table S1) for which sufficient expression data were available in the GeneSapiens database [18]. Expression levels of kinases were first analyzed across 55 major tumor types (n = 4078 samples) and 44 healthy tissues (n = 1603 samples) (Table S2). Expression of kinase genes across these 99 tissue types was first analyzed in a binary fashion, defining those tissues and tumor types where each kinase gene was transcriptionally active. Previously such binary level analysis has been shown to be a useful method to reduce noise [19], [20]. A kinase gene was defined to be transcriptionally active in a tissue if its median expression in the tissue was more than the predefined background expression level (Figure S1) across all healthy tissue types. We defined the background gene expression activity for each kinase across the 1603 samples representing the healthy human transcriptome (see methods section and Figure S1). Any kinase gene whose expression levels in healthy or tumor tissues exceeding this range were nominated as “transcriptionally active” (Figure 1, Figure S3) in the corresponding tissues. Thus, from this analysis we were able to define which tissue and tumor types most likely systematically expressed each kinase gene at an active level in majority of the samples of the tissue type and thus potentially contributing to the biological functions active in that tissue.

Clustering of the binarized expression levels of kinase genes along both the gene and tissue dimensions revealed distinct transcriptionally active kinome profiles and a classification of tissue types based on such activity profiles (Figure 1A–B). For example, kinome gene expression activity levels distinguished a group of “Healthy” tissues, of which 92.6% were normal tissues, with distinct subgroups of neuronal and muscular tissues. The group “Immunological and hematological” was almost entirely composed of (94.7%) immunological and hematological tissues. The “Solid cancer” group consisted of 94.7% of solid tumor samples, with subgroups of non-epithelial and epithelial cancers. Epithelial tumors could also be further divided into squamous and adenocarcinomas. Additionally, one mixed group was formed (50% healthy and 50% cancer).

Conversely, several groups of kinases having distinct transcriptional activity patterns across various tissue groups were identified (Figure 1A, Table 1). For example, one of the most prominent ones was mainly active in solid cancers and immunological/hematological tissues. Kinase genes belonging to this group were transcriptionally active in 88.7% of solid cancers and in 65.8% of immunological/hematological tissues. In the healthy and mixed tissue groups the percentages were 20.8% and 44.2%, respectively. This group of kinases was named “proliferation” kinase genes. Similarly, kinase genes mainly active in immunological/hematological tissues were identified (63.3% activity in these tissues vs, 11.3-16.7% in other tissue groups). Other identified example groups of kinase genes include “neuronal, ”non-epithelial”, “epithelial” and “generally” active (see Table 1 for average activity levels and Figure 1). Example kinases for the identified groups were *AURKA* [21] for proliferation kinases, *MATK* [22] for immunological/hematological kinases, *PRKCE* [23] for neuronal kinases, *PTK2* [24] for non-epithelial kinases, *ERBB2* [25] for epithelial kinases and RPS6KC1 [26] for general kinases. Measured mRNA expression levels for these example kinase genes are shown in Figure S2. Table S3 provides transcriptional activity information for all 459 kinases across 99 tissue types allowing further study of both individual kinase genes as well as healthy versus cancer comparisons between tens of tissue-malignancy pairs.

**Table 1:** Average percentage of tissues of each distinct tissue group where kinase genes of the identified groups are transcriptionally active.

Kinase group	Healthy	Healthy (neuronal)	Healthy (muscular)	Mixed	Solid cancer	Solid cancer (epithelial)	Solid cancer (non-epithelial)	Immunological
Proliferation	20.8%	12.5%	6.3%	44.2%	**88.7%**	**87.8%**	**87.1%**	**65.8%**
Immunological	14.5%	12.5%	13.5%	12.6%	16.7%	16.7%	11.3%	**63.3%**
Neuronal	26.2%	**55.4%**	25.6%	8.6%	5.4%	8.5%	9.7%	5.5%
Non-epithelial	32.3%	29.0%	28.6%	28.3%	32.2%	19.5%	**52.4%**	12.2%
General	**78.2%**	**70.0%**	**100%**	**75.0%**	**75.5%**	**84.0%**	**68.7%**	**52.6%**
Epithelial	36.9%	10.4%	12.5%	**57.1%**	37.7%	**58.8%**	9.4%	18.9%

### Co-expression network analysis to determine the functional context of kinase genes

**Figure 2:**
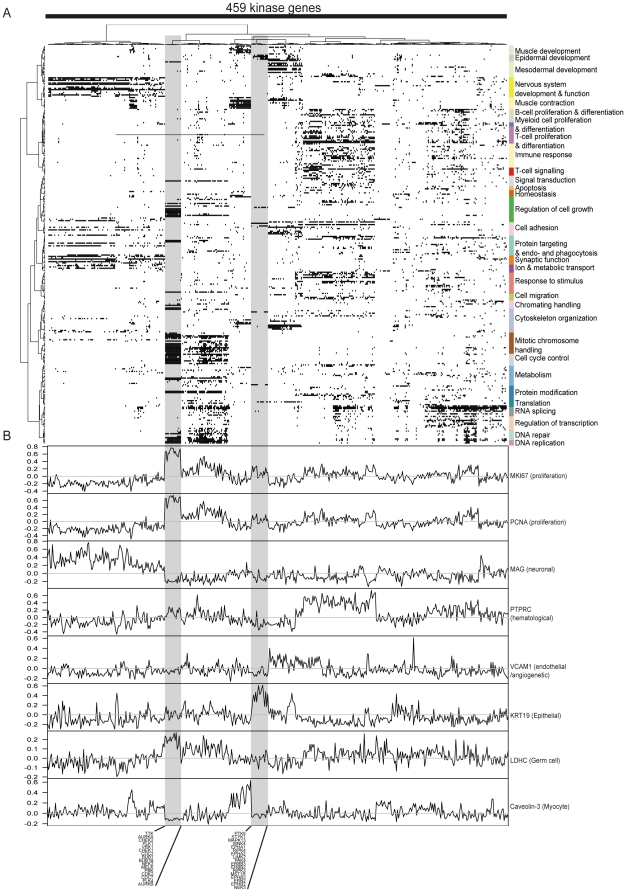
A) Functional associations of human kinase-encoding genes. The x-axis contains 459 kinase genes and the y-axis contains GO-BP classes. For the sake of clarity only those biological processes (GO-BP) enriched in the co-expression environment of at least 15 kinases are shown (301). Detailed information of all GO-BP class associations of the kinase genes are given in the Supplementary tables. The x-axis has been clustered with binary distance measure with complete linkage. The y-axis has been clustered in terms of semantic similarity of the GO-BP classes. The predominant biological interpretations of each cluster are given on the right side of the image. The analysis of the co-expression space made it possible to elucidate in what kind of biological processes kinase genes are expressed. **B) Pearson correlation coefficients of functional and tissue specific marker genes with the expression levels of each kinase gene.** Below the figure are listed the gene names in two groups kinase genes. The group on the left is associated with cell cycle and mitotic chromosome handling and has elevated correlation to *MKI67* and *PCNA*. The group on the right is associated to epidermal development and has elevated correlation to *KRT19*.

We then performed an analysis of the putative functional context associations for each kinase gene by defining their gene co-expression networks. This analysis was done with mRNA expression levels of kinases genes, not with the binarized data. The network of co-expressed genes around each kinase gene was calculated in a consecutive fashion, including up to a maximum of five co-expression links (see methods) originating from the kinase gene. Altogether, a total of 70.9 million correlations were processed. We then searched for statistically significant relative enrichments of Gene Ontology biological processes (GO-BP) [27] in the co-expression network around each kinase gene, resulting in putative functional context associations for each kinase gene (Figure 2, Figure S4). Complete information of all associations to each GO-BP class is given in the Table S4. Data for Figure 2A are given in the Table S5. Pearson correlation coefficients between the kinase genes and specific marker genes of well known biological functions were calculated to further validate the suggested functional associations of the kinase genes (Figure 2B). Expression of *MKI67* [28] and *PCNA* [29] genes, two well-established cell proliferation markers, showed the highest correlations with kinases strongly associating to mitosis and cell cycle. Similarly, *LDHC* (germ-cell specific marker) [30], *PTPRC* (marker for hematopoiesis) [31], *VCAM1* (endothelial/vascular cell marker) [32], K*RT19* (epithelial marker) [33], *MAG* (neuronal cell marker) [34] and *CAV3* (myocyte marker) [35] correlated with the kinase genes with corresponding functional associations.

**Figure 3 pone-0015068-g003:**
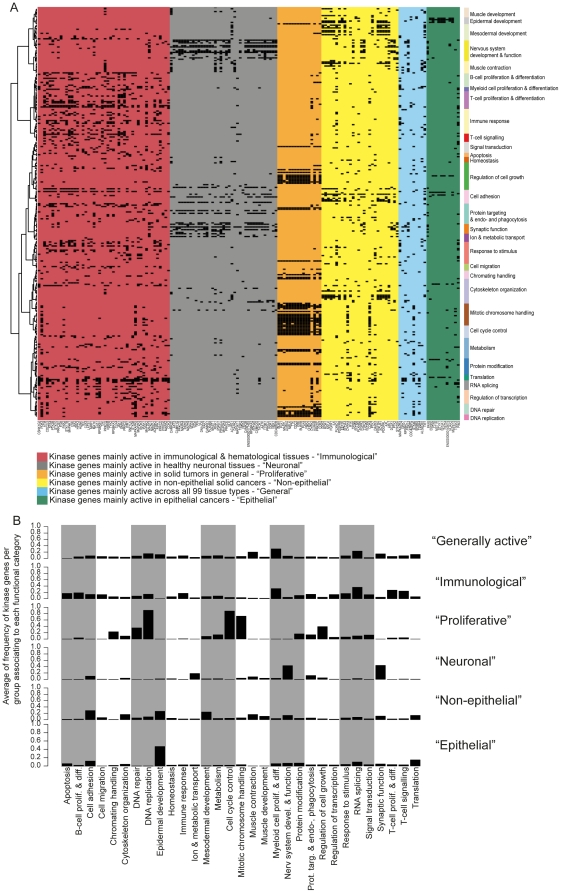
Functional context associations of example kinase gene groups (**Figure 1**). A) The y-axis contains GO-biological processes in the same order as in Figure 2. “Immunological” kinase genes (marked with red) associate mainly to B-cell, T-cell and myeloid cell proliferation & differentiation as well as to immune response. “Neuronal” kinase genes (marked with grey) associate strongly to neuronal functions. “Proliferation” kinase genes (marked with orange) associate very strongly to cell cycle control, mitotic chromosome handling, DNA replication, DNA repair and regulation of cell growth. “Non-epithelial” kinase genes (marked with yellow) associate most to cell adhesion, cytoskelton organization, epidermal development and mesodermal development. Functional associations of kinase genes active in most of 99 analyzed tissues (“General”, marked with blue) seems to cover almost all processes present in the analysis with marginally more in RNA splicing, muscle contraction and myeloid cell proliferation & differentiation. As assumed, “Epithelial” kinase genes associate strongly to epidermal development. B) The average frequency of kinase genes per group associating to each functional category.

We then elucidated the functional context associations of the kinase gene groups identified from the transcriptional activity data (Figure 1A, Table 1). Almost all kinase genes of the “proliferation” group (Figure 3) associated with DNA repair, cell cycle control, mitotic chromosome handling, chromatin handling and regulation of cell growth. These associations arise since the kinases were transcriptionally active in rapidly proliferating tissues (cancers and hematological tissues). These include the well-known mitotic kinase genes (*AURKA* [36], *BUB1* [37], *PLK1* [38], *TTK* [39], *CDC2* [40], *PBK* [41], *BUB1B* [42], *PLK4* [43], *NEK2* [44], *CHEK1* [45], *AURKB* [46], *CDK2* [46]), but also several novel ones (*MASTL, MELK, DYRK2, PRKDC*) which are not yet experimentally proven to be mitosis and/or cell cycle related. Similarly analysis of the “Nervous” kinase genes (Figure 3) gave associations with synaptic function, nervous system development & function. Kinase genes from “immunological” group had more diverse associations but included immune response, B-cell, myeloid cell and T-cell proliferation & differentiation, response to stimulus and RNA splicing (possibly related to heavy splicing activity of immunoglobulin genes). “Non-epithelial” kinase genes were associated with cytoskeleton organization, cell adhesion, mesodermal and epidermal development. Dominant functional context of “Epithelial” kinase genes was epidermal development. “General” kinase genes had associations with many diverse biological processes, suggesting a group of kinases with many different functions.

### Gains and losses of kinase activity in prostate and lung cancers

**Figure 4 pone-0015068-g004:**
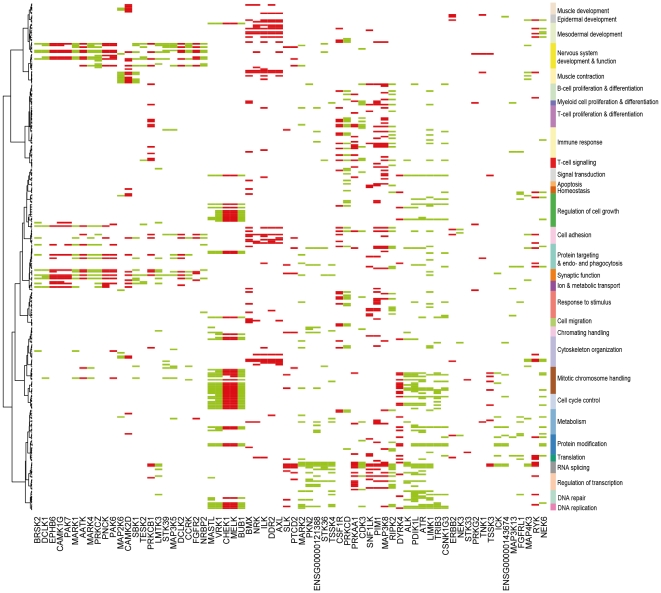
Gain and loss of transcriptional activity between healthy prostate and prostate cancer. On the x-axis (clustered with binary distance and Ward linkage) are 68 kinases whose transcriptional activity is either gained (green color) or lost (red color) in prostate cancer when compared to healthy prostate. On the y-axis are functional context associations of the kinases in the same semantically defined order as in Figure 2. This analysis allows identification of kinases whose transcription is elevated to active level or kinases whose biologically active level is most likely lost as well as the functional context to which the kinases are associated. Some notable changes in the kinome transcriptome include the losses of transcriptional activity of *BMX*, *NRK*, *ILK*, *DDR2*, *AXL* and *RYK* which all associate to processes like cytoskeleton organization, cell adhesion, meso- and epidermal development. Similarly, there is a group of kinases with gained transcriptional activity (*MASTL*, *VRK1*, *BUB1*, *ALK*, *PDIK1L*, *ATR*, *LIMK1*, *TRIB3*, *CSNK1G3*) associating to cell cycle control, mitotic chromosome handling, DNA replication and regulation of cell growth.

**Figure 5:**
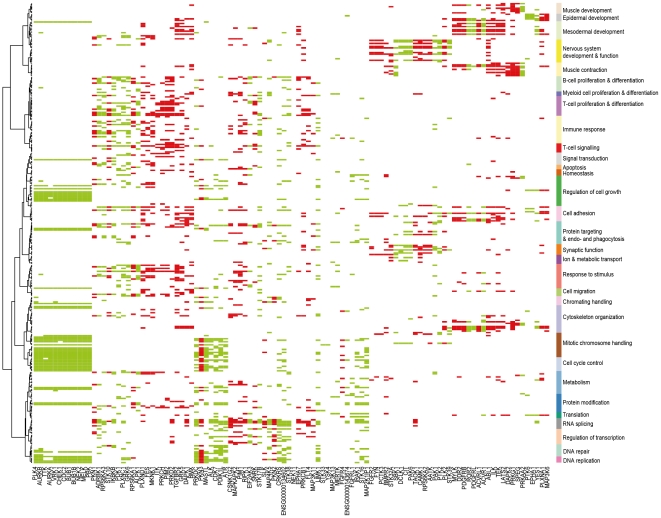
Gain and loss of transcriptional activity between healthy lung and lung adenocarcinoma. On the x-axis (clustered with binary distance and Ward linkage) are 106 kinases whose transcriptional activity is either gained (green color) or lost (red color) in lung adenocarcinoma when compared to healthy lung. On the y-axis are functional context associations of the kinases in the same semantically defined order as in the figure 2. This analysis allows identification of kinases whose transcription is elevated to active level or kinases whose biologically active level is most likely lost as well as the functional context to which the kinase genes are associated. Some notable changes in the kinome transcription include a major gain of kinases associating to DNA replication, cell cycle control, mitotic chromosome handling and regulation of cell growth.

After establishing the overall validity of interpretation of transcriptional activity levels (Figure 1A–B) and functional context associations (Figure 2A–B, Figure 3A–B) we studied which kinase genes gained and which lost transcriptional activity in malignant tissues as compared to the corresponding normal tissues (Figure 4, Figure 5) and how these cancer-related changes compared with the potential biological processes discovered for these genes. Comparing the transcriptional activity profile of healthy prostate with prostate cancer (PRCa) reveal that 37 kinase genes had gained and 31 lost transcriptional activity in malignant prostate cancers (Figure 4). This represents 14,8% of all the kinases indicating that for most kinases prostate cancers and normal tissues are rather similar, as reflected in the kinome clustering data (Figure 1). The kinase genes gaining activity in prostate cancer were associated with DNA replication, cell cycle control, mitotic chromosome handling and regulation of cell growth. Among these genes *BUB1* [37] is the best known to be mitosis related, but also *MASTL* had strong associations with mitosis and cell cycle. It does not have an experimentally proven role in mitosis except of one observation where it was recognized as part of the mitotic gene signature predicting poor survival in luminal breast cancer [47]. *CHEK1*, a kinase with a key role in maintaining genome integrity [48], and *MELK*, a kinase known to associate with embryogenesis and the undifferentiated state of cells [49], both lost their transcriptional activity in prostate cancer despite of being strongly associated with mitosis in the general gene co-expression network analysis. This indicates potentially important functions for these genes in prostate cancer. For example, the loss of *CHEK1* transcriptional activity may link to the recently reviewed deficient DNA repair process in PRCa [50]. The most prominent functional associations of kinase genes whose expression was lost in prostate cancer were linked to cytoskeletal organization, cell adhesion and mesodermal development (Figure 4). Among these genes *DDR2* [51] has previously shown to mediate contact inhibition of cancer cells.

Among the 37 kinase genes gaining activity in PRCa in comparison to healthy prostate there were 22 kinase genes (*MASTL*, *CCRK*, *NEK6*, *MAP3K13*, *DCLK1*, *CSNK1G3*, *ATR*, *SBK1*, *TESK2*, *BRSK2*, *FGFRL1*, *VRK1*, *PRKCZ*, *PKN2*, *LMTK3*, *CDK3*, *NRBP2*, *MAP4K3*, *MARK1*, *MARK4*, *TSSK4*, ENSG00000121388) without previous association to PRCa based on the Pubgene [52]. The remaining 15 kinase genes (*NEK3*, *STK39*, *LIMK1*, *TRIB3*, *STK36*, *BUB1*, *RIPK2*, *MARK2*, *MAP3K5*, *PRKCD*, ENSG00000143674, *MAP2K6*, *ALK*, *PDIK1L* and *ICK*) had 3–17 co-occurrences with PRCa in PubMed.

A similar comparison between healthy lung and lung adenocarcinoma revealed that 55 kinase genes gained and 51 lost transcriptional activity in the cancer, corresponding to a total of 23,1% of the studied kinase genes (Figure 5). This is reflected in the large difference of the transcriptionally active kinome between the healthy lung and lung adenocarcinoma (Figure 1B). Among the 55 kinase genes gaining transcriptional activity, the functional association to cell cycle control was found to be even more prominent than in PRCa (Figure 4). *EGFR* was found to be transcriptionally active in very many epidermal tissues, including healthy and malignant lung tissues, which is as expected from one of the most influential epithelial growth factor receptors. *ALK* is also among the kinases that gain activity in lung adenocarcinomas, lung carcinoid tumors and squamous cell lung carcinomas in comparison to healthy lung. Its main functional associations relate to DNA repair, DNA replication, mitotic chromosome handling and mRNA splicing.

### Exploration of poorly known kinases genes for their transcriptional activity and functional context associations

**Figure 6:**
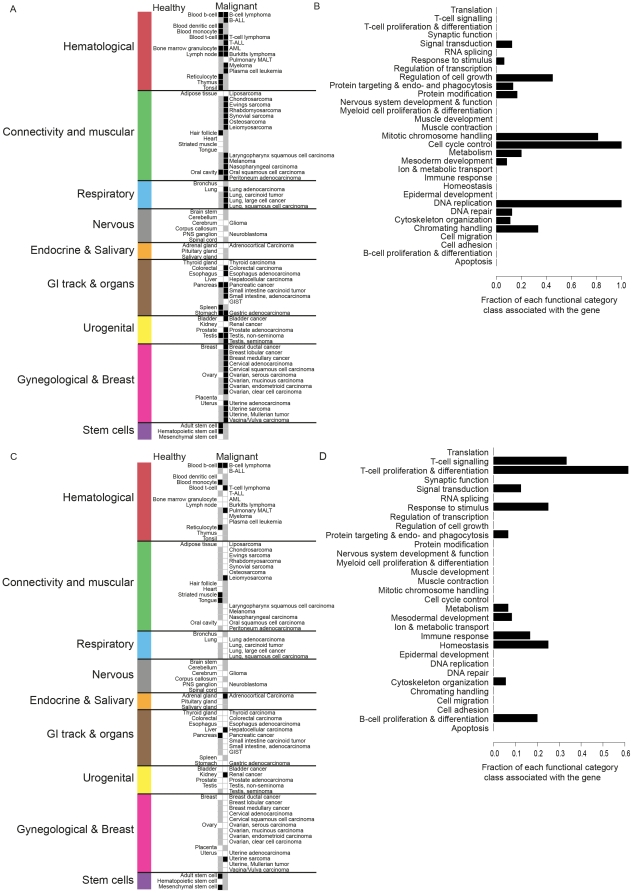
Transcriptional activity level across 44 healthy and 55 malignant tissues and functional context associations of individual kinases. A) Transcriptional activity levels of *VRK1*. Vertical ordering of tissues is based on the anatomical system from which the tissue originates (colored bar on the left). The left column shows transcriptional activity levels across 44 healthy tissues (white  =  transcriptionally non-active, black  =  transcriptionally active). The right side shows transcriptional activity levels across 55 malignant tissues. B) Functional context associations of the *VRK1*. Barplot shows the fraction of GO-BP classes of each functional category being associated to the gene through its co-expression environment. According to the analysis *VRK1* seems to be in generally transcriptionally active in both healthy and malignant hematological tissues. It is also active in almost all tumors of connectivity and muscular system (sarcomas, head and neck and melanoma). The most prominent difference between healthy and malignant tissues is in female specific tissues as the gene is transcriptionally active in all histological subtypes of breast, cervical, ovarian and uterine cancers, but not in any of the corresponding healthy tissues. *VRK1* has strong functional context associations to cell cycle control, mitotic chromosome handling and chromatin handling. C) C21orf7 is transciptionally active in immunological tissues, especially in several lymphomas. It is also active in mesenchymal and adult stem cells. Additionally, there is a possible ectopic expression of this otherwise lymphoid and stem cell specific gene in few distinct carcinomas. D) The functional context associations lands gene firmly to the B- and T-cell signaling and differentiation as well as to immuno response, response to stimulus and homeostasis related processes.

Transcriptional activity levels and functional context associations make it possible to explore and annotate the entire kinome, and hence provide potential starting points to predict context-specific functions of relatively poorly understood kinase genes. For example, *VRK1* was found to be transcriptionally active in both healthy and malignant hematological tissues (Figure 6A). Previously it has found to differentiate Imatinib responders among CML patients [53], but otherwise its role in hematological tissues is not well known. It is also active in almost all tumors of connective and muscular system (sarcomas, head and neck and melanoma), with a previously shown role in head and neck squamous cell carcinomas [54]. The most prominent difference in transcriptional activity between healthy and malignant tissues for VRK was seen in gynecological cancers, including breast, cervical, ovarian and uterine cancers. *VRK1* had strong functional context associations to cell cycle control, mitotic chromosome handling and chromatin handling biological processes and it has recently been associated with mitosis and performs similar functions as *AURKA* [54], [55], [56] and its role in cell proliferation has been shown with siRNA experiments [57] (Figure 6B). Previously, *VRK1* had not been associated with all of these cancers even though it is part of the signature depicting poor survival of luminal breast cancer patients [47].

An almost completely unknown kinase gene from an open reading frame of chromosome 21 was C21orf7 (also known as *TAK1L*), which we decided to study in more detail. C21orf7 was found to be transcriptionally active in immunological tissues, especially in several lymphomas and in mesenchymal- and adult stem cells (Figure 6A). This corresponds to what is previously known about this gene, an association to differentiating stem cells [58] and strong expression in peripheral leukocytes [59]. Additionally, it was found to be transcriptionally active in adrenocortical-, renal- and hepatocellular carcinomas. The functional context associations link this gene firmly in the B- and T-cell signalling and differentiation as well as to immunoresponse, in line with a prominent transcriptional activity in immunological tissues (Figure 6D). The functional annotations also link this gene with signal transduction, metabolism, mesodermal development and cytoskeleton organization, perhaps elucidating some of the biological processes to which the gene associates in stem cells [58].

Another relatively unknown kinase gene *IGFN1* (ENSG00000163395) is previously known only to down-regulate protein synthesis during the denervation of skeletal muscle as well as to provide structural support for the skeletal muscle sarcomere [60], [61]. Our analysis revealed it to be transcriptionally active in various healthy tissues like pancreas, testis, striated muscle, ovary and uterus. Additionally, lung carcinoid tumor had transcriptional activity without activity in the most corresponding healthy tissue. Functional context associations reveal this kinase gene to be mainly related to muscle contraction, muscle and heart development, epidermal development, translation, skeletal cytoskeleton organization and cellular calcium ion homeostasis (Table S3, S4 and S5). Interestingly, there are previous indications that neuroendocrine carcinomas have a higher tendency to differentiate towards skeletal muscle [62], [63], perhaps explaining the observed transcriptional patterns in the lung carcinoid tumor.

## Discussion

We assessed the transcriptional activity levels of human kinase genes across 99 tissues and tumor types bioinformatically, and were able to show how the transcriptionally active kinomes are distinct from one tissue type to another and between cancer and normal tissues. While there are hundreds of studies on the expression of individual kinase genes or proteins in specific sample types, this represents to our knowledge the first systematic attempt to compare mRNA expression levels across all kinases and across all major tissue and tumor types with similar methodology. Based on the definition of the transcriptionally active kinome, we observed five broad categories of tissue types, including hematological-immunological, other normal tissues, as well as epithelial and non-epithelial cancers (Figure 1B). Epithelial cancers were further divided into squamous- and adenocarcinomas. These observations characterize expression activities of the kinase genes systematically across the full spectrum of normal and malignant diseases. The results suggest that the transcriptional activities of kinases can cluster tissue types in a biologically meaningful way, despite the fact that the activity of kinases is strongly regulated by post-translational events.

We also estimated the biological context of kinase genes from the analysis of their transcriptional co-expression environment [64], [65]. Biological processes that were linked to specific actively transcribed kinases included immune response, neuronal, cell proliferation, mRNA translation and muscle function. The group of “proliferation” associated kinase genes was linked to cell cycle control, mitotic chromosome handling, DNA replication, DNA repair and regulation of cell growth whereas example kinase genes associating to these processes were well known mitosis related kinases *AURKA* [*21*], *BUB1* [37] and *TTK* [*66*]. “Neuronal” kinase genes were associated to GO-terms nervous system development & differentiation and synaptic function whereas example kinase genes associating to these processes were *PRKCE* [*23*] and *EPHA4* [67], both known for nervous system related functions. Functional context associations arising from gene co-expression analysis do not suggest new GO annotations for genes. However, they reveal the biological processes of the poorly known kinase genes based on the known functions of other genes that are coordinately expressed with the gene of interest. The method of co-expression analysis used here finds systematic functional associations that are consistent and shared across the different tissue and tumor types. There could be additional highly tissue-specific functions that could be identified using co-expression analysis within a specific tissue/tumor type. Overall, classification of kinases based on transcriptional activity and functional associations complements the established structural homology classification by Manning et al.[68]. Importantly, there is very little similarity based on kinome protein structure and the expression patterns of the kinases indicating that knowing these two properties of kinases is important for understanding the biological and therapeutic implications of this group of genes.

For example, a mitosis related kinase gene *VRK1* was found to be active in many hematological tissues as well as in the many tumors of connective and muscular tissues (sarcomas, head & neck and melanoma) and in most gynecological cancers (uterine, ovarian and breast cancers) (Figure 6A–B). Functional associations reflected what is already known about *VRK1*, like its association to cell cycle control and mitosis with similar functions as *AURKA* [54], [55], [56]. It has also been established that *VRK1* and p53 form an autoregulatory loop where active wt-p53 inhibits *VRK1* but altered p53 is unable to do so [71].

C21orf7, also known as *TAK1L* and TGF-beta activated kinase like gene is a rather poorly known kinase gene [58]–[59]. Its name originates from sequence homology with *TAK1*, which belongs to the nuclear hormone receptor family. However, there is no information regarding functional similarities between *TAK1L* and *TAK1*. Analysis of its transcriptional activity confirmed TAK1L to be a hematological- and stem cell-specific gene with gene co-expression analysis associations indicating potential involvement in T- and B-cell differentiation, immune response as well as homeostasis and mesodermal development (Figure 6C-D). *TAK1L* has been previously reported to be one of the genes overexpressed in mesenchymal stems cells during osteogenic differentiation [58] and having highest expression in peripheral leukocytes [59]. Interestingly, we observed transcriptional activity in some solid tumors, such as uterine sarcoma, renal-, adrenocortical- and hepatocellular carcinomas, with little or no expression in the corresponding normal control tissues. This could reflect a role for this gene in tumor progression, perhaps via de-differentiation and tumor stem cell involvement.

Analysis of *IFGN1* revealed known functional roles for this gene, including relation to muscle development and contraction as well as protein translation [60], [61]. However, the co-expression environment analysis gave additional information that this kinase gene may also play a role in epidermal development, keratinization in particular. Systematic analysis of its transcriptional activity also revealed that it is active in various other healthy tissues like pancreas, testis, ovary, uterus and striated muscle as well with a potential role in carcinomas with neuroendocrine differentiation like in lung carcinoid tumor.

As this study provides information on the systematic transcriptional activity levels of all kinases in all tissues, as well as functional associations of each kinase, we have released all the data on tissue and disease links as well as predicted functional roles of the kinases in the supplementary data to support the utilization of these insights by the scientific community interested in specific kinases (Table S3, S4 and S5).

The transcriptional levels of the kinase genes as well as their predicted functional associations are likely to be essential when exploring the role of kinases in disease and when defining indications where kinase activity in a disease tissue is higher than that in any normal tissue. This could provide a basis for specific therapeutic targeting.

The kinase genes gaining transcriptional activity in PRCa when compared to healthy prostate were associated with cell cycle control, mitotic chromosome handling and DNA replication; while kinase genes were losing transcriptional activity in PRCa were associated with cytoskeleton organization, cell adhesion, meso- and epidermal development. Altogether 22 kinase genes previously not associated to PRCa were shown to be transriptionally active in PRCa but not in healthy prostate. These kinase genes include *CCRK* which is established cell cycle related kinase [69], *MASTL* which is a relatively unknown kinase gene associated with mitosis through co-expression analysis and *NEK6* which is also an established mitosis related kinase [70]. Changes between healthy lung and lung adenocarcinoma (Figure 5) were more prominent than in the case of prostate suggesting that lung cancer progression involves a deeper deregulation of its kinome transcriptome than in prostate cancer (B).

Also, systematic analysis of kinase transcriptional activities across all healthy tissues could help to prioritize for further study those kinases, whose activation is most disease specific, and hence whose inhibition would theoretically cause fewer side effects. For example, *EGFR* was found to be transcriptionally active in healthy skin (hair follicles), adrenal gland, bladder, lung, esophagus and colorectal, bronchus, kidney, mesenchymal and adult stem cells in addition to CNS and some gynecological tissues. Currently identified side effects of the various anti-*EGFR* therapies include skin rash due to the changes in keratinocyte and hair follicle maturation [72], interstitial lung disease and other respiratory problems [72], [73]. Gastrointestinal toxicity [73] and hypomagnesemia have been reported due to the *EGFR* blockade in kidneys [72]. These correspond well with the observed healthy tissues having a transcriptionally active *EGFR. ERBB2/HER-2* was found to be transcriptionally active in various healthy tissues, including heart, colorectal, esophagus, kidney, bladder, bronchus and lung. The most common adverse effects of anti-ERBB2 therapy are cardiotoxicity and intestinal problems like diarrhea [73] with some indications of respiratory problems [74], [75]. Obviously, there are many reasons for side effects in the human body, and these cannot be reduced simply to transcriptional levels of the genes. Nevertheless, these examples give some indication of the potential of this transcriptomics approach to predict normal tissue effects of kinase inhibitors.

Results presented in this paper rely to a large extent on the GeneSapiens database [18] (www.genesapiens.org), which provides integrated gene expression data and gene co-expression environment analysis [18], [76]. Transcriptional activity levels of kinases were estimated by taking advantage of the genome-wide data coverage of GeneSapiens to determine the overall body-wide background levels for the transcriptional activity of each kinase gene. This is a distinct advantage of the present method, which takes into account the transcriptional variability of genes across the body, not just between e.g. a cancer of one organ and the corresponding normal tissue as is typically done in biomedical studies. Systematic use of integrated gene expression data, such as GeneSapiens, allows the definition of universal cutoff points for transcriptional activity across all tissue types, which then makes it possible to identify active gene expression regulation in a tissue even when the relative expression increase between tissues to be compared is only modest. Human tissues have distinct transcriptionally active kinomes with functional associations supporting the results. Most of the known kinases were active in previously reported tissue types and had expected functional associations. The present study was designed for systematic global characterization of kinase gene transcriptional activity and functional context associations, therefore it is not optimal to directly pinpoint the most obvious therapeutic targets of each cancer type.

Expression levels were binarized (on/off) for the global comparisons across all tissue and tumor types in order to facilitate computations, data interpretation and to reduce noise [19], [20]. The co-expression environment was analyzed from the actual measured mRNA expression levels, not from binarized data. Co-expression analysis using Pearson correlations has been previously shown to be a useful technique to facilitate understanding of gene functions [64], [65], [77]. Our present analysis focused on generic co-expression associations. An interesting future aspect for co-expression environment analysis is to define the gene co-expression network and subsequent functional associations in a tissue-specific manner. This would provide more specific functional associations and allow more detailed understanding of functions related to chances in transcriptionally active kinome between e.g. healthy and malignant counterparts. Obviously, any bioinformatic estimation of the transcriptomic activity is highly dependent on the thresholds applied, and laboratory validation of the results is needed. Original non-binarized gene expression data of all presented kinases can readily be explored at www.genesapiens.org. Example figures of bodywide expression levels of specific kinases, including *AURKA*, *PTK2*, *MATK*, *ERBB2*, *PRKCE* and *RPS6KC1*, which were discussed above, are shown in Figure S2.

In summary, we have shown how the definition of transcriptional activity of kinases and their co-expression environment will help identify potential functional roles of the kinases in health and disease. To our knowledge this is first systematic characterization of the human kinome across major human tissue and cancer types at the transcriptional level, together with functional associations with other transcribed genes. Major tissue classes having a distinct transcriptionally active kinome were found to be 1) healthy tissues, with subgroups of neuronal and muscle tissues, 2) immunological/hematological tissues, 3) solid tumors with subgroups of epithelial and non-epithelial tumors and 4) a mixed class. The most readily indentified functional associations for a group of kinases included proliferation related processes, neuronal process, muscle tissue processes, DNA replication and repair, transcription and translation regulation, immunological response and development processes of various tissue types. This “body-wide” approach for transcriptomic analysis of gene activity and functional context could readily be expanded to other biologically and medically interesting gene sets.

## Methods

### Definition of kinase genes

Human protein kinase genes were selected from the Panther database version 6.1 [78], [79]. This set of protein kinases (529) was further filtered in terms of expression data availability from GeneSapiens database [18] for a total of 459 protein kinases (Table S1). Kinase genes had a minimum of 2583 common values with each other over 5681 samples covering 99 distinct healthy and pathological tissues (Table S2).

### GeneSapiens database

Expression data were fetched from the GeneSapiens database [18] with additional data from GEO studies GSE15459, GSE12452, GSE9843, GSE10927, GSE8167, GSE9576, GSE12102, GSE13314 and GSE9844 to extend the tissue coverage of the GeneSapiens data. GeneSapiens contains integrated gene expression data from 9783 samples covering 175 types of healthy and pathological human tissues. Data in GeneSapiens have been integrated and normalized as described by Kilpinen et al. [18] and Autio et al. [76]. Additional data were integrated by using MAS5 and EQ normalization as previously described [18], [76] and AGC normalization [18], [76] by using gene and array specific correction factors used in GeneSapiens database construction [18]. This process rendered the new data directly comparable with existing GeneSapiens data.

### Definition of active transcription

Expression levels of each gene were analyzed to define tissues where it is actively transcribed. In other words, each gene was tested in each tissue to see whether its expression level was above the defined background noise levels. The gene was defined to be transcriptionally active in a tissue if its median expression in the tissue was more than the predefined background expression level (Figure S1). Background expression level for each gene was calculated as follows: expression level entropy of sample annotation class labels was calculated in a sliding window (with width of 5% of maximum expression value) from zero expression to maximum level. The midpoint of the window with the highest entropy level was recorded. The variance of the data below this midpoint was calculated, and 2 x standard deviation was added to the midpoint to reach the background cutoff level. 2 x SD results in 95% coverage of the assumed normal distribution of the background expression the gene. Thus, every gene defined as transcriptionally active has a p-value ≤ 0.025 against the null hypothesis that it derives from the background expression (which should be not be defined as transcriptionally active). Selection of a higher threshold leads to more stringent conditions of defining genes as transcriptonally active, which eventually degrades the resolution of the digitalized expression levels' ability to separate tissues. 2 x SD was found to be reasonable compromise between reliability and sensitivity.

### Co-expression network analysis

To define functional context associations of kinase genes we calculated a genomic co-expression network across 5712 samples (31 samples included here were omitted from transcriptional activity level calculations due to the incomplete annotation) for each of the kinase genes. The genes (n = 11 906) were chosen so that all genes had enough values in common to calculate correlation coefficient. We then calculated Pearson correlation coefficients between all genes. The correlation network around each of the kinases was then analyzed to identify prominent genes related to the kinases. This analysis was based on random walking along defined correlation links (edges) between genes (nodes). For each kinase, we performed 500 random walks, each 5 steps long, originating from the kinase in question, collecting all genes (nodes) encountered. Steps were not allowed to go directly backwards and each step was allowed to randomly choose only from valid correlation links. The validity of correlation links was defined on a gene-by-gene basis as follows. Valid links for each gene were those having a correlation value in the top 99.9 percentile of all correlations for that gene. This was done since the variability of correlation coefficients hindered efforts to define a single universally applicable cutoff level. Thus, for each kinase we identified a frequency distribution of genes that were encountered in the near vicinity at its co-expression network. Overall, this co-expression network analysis method took into account the topology of co-expression network and the highly dynamic range of correlation coefficients between the genes. Genes identified in the co-expression network of each kinase were subsequently analyzed in terms of Gene Ontology biological process (GP-BP) class enrichments.

### Functional context associations of kinase genes

Genes in the co-expression network around each kinase were analyzed for significant enrichments of GO-BP classes by using R library GOSim [80]. All enrichments with p-value <0.01 were accepted. This analysis resulted in a list of significantly enriched GO-BP classes in the co-expression network around the kinase, thus associating each kinase with GO-BP classes. For visualization, only those GO-BP classes associating with at least 15 kinase genes were selected (301 GP-BP classes). The order of GO-GP classes (y-axis) in Figures 2–5 is defined by clustering GO-BP classes by using semantic similarity between the GO-BP classes over the entire diacyclic graph (DAG) of GO-BP ontology (GOSim library [80], Lin semantic similarity measure [81] and Ward linkage method).

## Supporting Information

Figure S1 **Schematics of defining transcriptionally active level of gene (** ***ERBB2*** **shown as an example).** A–B entropy of tissue type distribution is calculated in a sliding window (window width 5% of the maximum of the gene) and expression level with maximum entropy is identified C) standard deviation of data points below the identified level is calculated D) Background level is defined to be 2 x standard deviation + the expression level with maximum entropy. The gene is defined to be transcriptonally active in a tissue if the median of tissue is above the background activity level.
(EPS)

Figure S2 **Bodywide expression profiles of six example kinases from GeneSapiens.** Green boxes are healthy tissues while red boxes are malignant tissues. Median expression level of the gene in question is indicated by black line, boxes extend from 25^th^ to 75^th^ percentiles, while whiskers extend to the 1.5*IQR. Data points beyond are shown as individual points. A) AURKA shows generally increased expression in cancers and in some proliferation active healthy tissues B) PTK2 is mainly expressed in mesenchymal and neuronal tissues highlighting the non-epithelial classification of the kinase gene C) MATK is expressed in immunological/hematological tissues D) ERBB2 is expressed mainly in epithelial tissues E) PRKCE is relatively specifically expressed in both central- and peripheral nervous system F) RPS6KC1 is expressed in various healthy and malignant tissues.
(EPS)

Figure S3 **Zoomable eps version of the** **Figure 1** **A) Kinase transcriptional activity over 44 healthy and 55 malignant tissues.** The number of samples per tissue is given in parentheses. Black indicates transcriptional activity of the kinase in the tissue. Figure has been clustered in both dimensions (binary distance measure with complete linkage). Several tissue groups can be identified (marked as color bars on the right side of the image). Correspondingly several groups of kinases can be identified having distinctly different activity profile in tissue groups (colored vertical bars). B) Tree of tissues as defined by transcriptionally active kinome (same as on the left side of image in panel A). Four main groups of tissues are mainly solid healthy tissues (92.6%), immunological & hematological (94.7%), solid cancer tissues (94.7%) and mixed one. Within these groups there are some more specific clusters like neuronal and muscular in healthy side and non-epithelial and epithelial on the cancer side. Epithelial cancers also show visible tendency to cluster to adeno and squamous groups according to their transcriptionally active kinome.
(EPS)

Figure S4 **Zoomable eps version of the** **Figure 2** **A) Functional associations of human kinase-encoding genes.** The x-axis contains 459 kinase genes and the y-axis contains GO-BP prosesses. For the sake of clarity only biological processes (GO-BP) enriched in the coexpression environment of at least 15 kinases are shown (301). X-axis has been clustered with binary distance measure with complete linkage. Y-axis has been clustered in terms of semantic similarity of the GO-BP classes. The predominant biological interpretations of each cluster are given on the right side of the image. The analysis of the coexpression space made it possible to elucidate in what kind of biological processes kinase genes are expressed. B) Pearson correlation coefficients of functional and tissue specific marker genes with the expression levels of each kinase.
(EPS)

Table S1 (TXT)

Table S2 (TXT)

Table S3 (TXT)

Table S4 (TXT)

Table S5 (TXT)
